# Exposure of *E. coli* to DNA-Methylating Agents Impairs Biofilm Formation and Invasion of Eukaryotic Cells via Down Regulation of the N-Acetylneuraminate Lyase NanA

**DOI:** 10.3389/fmicb.2016.00147

**Published:** 2016-02-11

**Authors:** Pamela Di Pasquale, Marianna Caterino, Angela Di Somma, Marta Squillace, Elio Rossi, Paolo Landini, Valerio Iebba, Serena Schippa, Rosanna Papa, Laura Selan, Marco Artini, Anna Teresa Palamara, Angela Duilio

**Affiliations:** ^1^Department of Chemical Sciences, University of Naples “Federico II” Naples, Italy; ^2^Department of Molecular Medicine and Medical Biotechnology, University of Naples “Federico II” Naples, Italy; ^3^Department of Biosciences, University of Milan Milan, Italy; ^4^Department of Public Health and Infectious Diseases, Pasteur Institute Cenci, Bolognetti Foundation, Sapienza University of Rome Rome, Italy; ^5^Department of Public Health and Infectious Diseases, Sapienza University of Rome Rome, Italy; ^6^IRCCS, San Raffaele Pisana Telematic University Rome, Italy

**Keywords:** DNA alkylation, adaptive response, NanA lyase, comparative proteomics, biofilm formation, virulence, adherent invasive *Escherichia coli* (AIEC)

## Abstract

DNA methylation damage can be induced by endogenous and exogenous chemical agents, which has led every living organism to develop suitable response strategies. We investigated protein expression profiles of *Escherichia coli* upon exposure to the alkylating agent methyl-methane sulfonate (MMS) by differential proteomics. Quantitative proteomic data showed a massive downregulation of enzymes belonging to the glycolytic pathway and fatty acids degradation, strongly suggesting a decrease of energy production. A strong reduction in the expression of the N-acetylneuraminate lyases (NanA) involved in the sialic acid metabolism was also observed. Using a null NanA mutant and DANA, a substrate analog acting as competitive inhibitor, we demonstrated that down regulation of NanA affects biofilm formation and adhesion properties of *E. coli* MV1161. Exposure to alkylating agents also decreased biofilm formation and bacterial adhesion to Caco-2 eukaryotic cell line by the adherent invasive *E. coli* (AIEC) strain LF82. Our data showed that methylation stress impairs *E. coli* adhesion properties and suggest a possible role of NanA in biofilm formation and bacteria host interactions.

## Introduction

All living organisms are exposed to alkylating conditions caused by both endogenous (metabolites, ROS, free radicals, etc.) and exogenous (pollutants, chemoterapics, drugs, etc) species. Biological macromolecules including DNA, RNA, proteins and lipids are sensitive to electrophilic species permeating cellular defenses and then subjected to alkylation damages (Fu et al., 2012). Due to its long biological half-life, its presence in a single copy and its functional role, the DNA is the most sensitive target for methylation, which can have strong repercussions on cell survival (Jena, 2012). Methylating agents, like methyl methanesulphonate (MMS), comprise a major class of DNA-damaging compounds that occur both endogenously and in the environment (Lobo et al., 2010).

To counteract the effects of DNA alkylation, all organisms evolved multiple DNA repair strategies, which can either respond to the DNA lesions or rely on sensing of the alkylating agents. The occurrence of either event is recognized by specific regulators that activate transcription of several genes to provide a definite response (Volkert and Landini, 2001). In bacteria, fluctuating levels of environmental alkylating agents activate an inducible response that enhances cellular resistance to the same agents. This adaptive response has been studied most extensively in *Escherichia coli*, in which induced alkylation resistance results from increased expression of four genes, *ada, alkB, alkA*, and *aidB* (Landini and Volkert, 2000; Rippa et al., 2010).

Besides the induction of the Ada-related adaptative response, little is known on the global effect of methylation stress on *E. coli*, that greatly affects cells survival (Rippa et al., 2011). A comprehensive investigation of *E. coli* response to alkylating agents was then pursued in an attempt to identify the most affected cellular pathways. Differential proteomics experiments were designed to evaluate the changes in the proteomic profiles in the presence and in the absence of methylating molecules.

Quantitative proteomic data showed a large decrease in the expression of proteins involved in energy production pathways. However, the most intriguing result was the massive downregulation of the N-acetylneuraminate lyases NanA, an enzyme involved in sialic acid metabolism. Using different biological assays we demostrated that impairment of NanA expression greatly reduced biofilm formation and *E. coli* adhesion to HeLa cells. Moreover, inhibition of NanA activity by a synthetic drug strongly decreased adhesion and invasion to Caco-2 cells of the intestinal pathogen *E. coli* LF82 (AIEC). Our results suggest a possible role of *E. coli* lyase NanA in biofilm formation and in interaction with eukaryotic cells.

## Methods

### *E. coli* strains

*E. coli* MV1161, a derivative of the standard laboratory strain AB1157, was used as reference strain in this work. Isogenic Δ*nanA::kan* mutant was constructed using the λ Red technique (Datsenko and Wanner, 2000). The MV1161 Δ*nanA::kan* mutant was transformed with pMAL-c5x-*nanA* plasmid containing the *nanA* gene in order to complement lyase activity. Both the Δ*nanA* mutant and the complement strain showed a growth rate superimposible with the parental strain. The strain LF82 (Boudeau et al., 1999), a prototype adherent-invasive *E. coli* (AIEC) strain, was used in adhesion and invasion experiments with Caco-2 eukaryotic cell line (http://www.lgcstandards-atcc.org/products/all/HTB-37.aspx?geo_country=it). LF82, was grown overnight in LB medium at 37°C, 180 rpm agitation, then used to inoculate 1:100 two different 250 mL flasks containing 100 mL of 22°C pre-warmed LB and underwent growth at 37°C, 180 rpm. Once reached an OD_600_ = 0.5, MMS was added to one flask at 0.04%, and OD_600_ measurements were taken every h from both flasks.

### Differential in gel electrophoresis (DIGE)

DIGE experiments were performed on four biological replicates of MMS treated and four biological replicates of MMS untreated *E. coli* as previously described (Caterino et al., 2009). *E. coli* cells were homogenized in 0.5 ml of lysis buffer (7 M urea, 2 M thiourea, 4% chaps, 30 mM Tris-HCl pH 7.5) using a Dounce homogenizer. Concentration of the protein extracts was determined and equal amounts of protein lysates were then labeled *in vitro* using two different fluorescent cyanine minimal dyes (Cy3 and Cy5, respectively) differing in their excitation and emission wavelengths. A third cyanine dye (Cy2) was used to label a mixture of all samples as internal standard. The three differently labeled protein mixtures were pooled and subjected to isoelectric focusing on a 3–10 pH range with 24 cm strips using an IPGphor 2 (GE Healthcare) at 20°C (costant voltage 300 V for 3 h, voltage gradient to 1000 V in 6 h, voltage gradient to 8000 V in 3 h and costant voltage 8000 V for up to 4 h). The current limit was 50 mA/strip. The samples for the semipreparative gels were focused in a separate run using the same IEF condition. Following IEF, strips were equilibrated in 100 mM Tris, pH 8.0, 30% v/v glycerol, 2% w/v SDS, 6 M urea, reduced with 0.5% w/v DTT for 15 min and alkylated with 4.5% w/v iodoacetamide for further 15 min. IPG strips were then transferred to the top of a classical 12.5% SDS PAGE gel for a second orthogonal electrophoresis analysis. The SDS PAGE separation was performed at 2 W/gel, for 16 h at 25°C.

The Cy2, Cy3, and Cy5 images were obtained by scanning each of the four DIGE gels at excitation/emission wavelength of 480/530 nm for Cy2, 520/590 nm for Cy3 and 620/680 nm for Cy5 using a Typhoon 9410 TM scanner (GE Healthcare). The semi-preparative gel, prepared in an identical fashion, was scanned with 480/633 nm wavelengths. After consecutive excitation at both wavelengths, the images from the preparative gel were overlaid and subtracted (normalized) from the samples, whereby only differences (up or down expressed proteins) between the two samples were visualized. DeCyder software 5.01 (GE Healthcare) was used to compare the abundance changes between the control and treated samples. The Batch Processor Module (GE Healthcare) was used for normalization of the signals from each CyDye channel and spot detection on the 12 images from the four DIGE gels and the calculation of the volume ratio for each spot pair (Cy3:Cy2 and Cy5:Cy2 ratios). The Biological Variation.

Analysis (BVA) module (GE Healthcare) was used to match all the 12 spot maps from the four DIGE gels and calculate the average abundance changes and paired Student's *t*-test *p*-values for the variance of these ratios for each protein pair across all four samples.

By performing a high resolution image analysis on the four biological replicates, it was possible to visualize significant differences between numerous protein spots present on the gels. Differential spots were defined as having a variation higher than 1.2 (*p* < 0.05) per previously established methods (Cella et al., 2012). The gels showed a high degree of similarity, with more than 80% of all spots superimposeable. The remaining 20% showed variation and were further studied.

### Proteomic analysis

The spots of interest were excised, hydrolyzed and the peptide mixtures analyzed by MALDI-MS and LC-MSMS mass spectrometry, on a 4800 Plus MALDI TOF/TOF™ (Applied Biosystems, Framingham, MA, USA) and a LC/MSD Trap XCT Ultra (Agilent Technologies, Palo Alto, CA) equipped with a 1100 HPLC system and a chip cube (Agilent Technologies) respectively. MALDI spectra were acquired in the positive ion reflector mode using delayed extraction in the 800–4000 Da mass range. LC-MS/MS analysis was performed using data-dependent acquisition of one MS scan followed by MS/MS scans of the three most abundant ions in each MS scan. Raw data analyses were converted into a Mascot format text to identify proteins using Matrix Science software. The protein search considered the following parameters: non-redundant protein sequence database (NCBInr), specificity of the proteolytic enzyme used for the hydrolysis (trypsin), taxonomic category of the sample, up to one missed cleavage, cysteines as S-carbamidomethylcysteines, unmodified N- and C-terminal ends, methionines both unmodified and oxidized, putative pyro-Glu formation by Gln, precursor peptide maximum mass tolerance of 200 ppm, and a maximum fragment mass tolerance of 200 ppm.

Identified proteins were investigated to predict functional protein association networks for each entry using the STRING online database (http://string-db.org). Proteins were analyzed with the STRING software.

### RNA extraction, reverse transcription and real-time PCR

Three *E. coli* culture replicates were grown at 37°C in the presence and in the absence of 0.04% MMS according to proteomics experiments. In order to obtain an immediate stabilization of RNA, two volumes of RNA protect Bacteria Reagent (Qiagen) were added directly to one volume of bacterial culture (10 OD600 nm). Bacterial cells were harvested and each pellet was stored at -20°C overnight. Total RNA was extracted from culture using RNasy Midi Kit (Qiagen) according to the manufacturer's instructions. The purity and integrity of total RNA was verified by both 1% denatured agarose electrophoresis and by evaluating that the A260/A280 absorbance ratio was within the range 2.1–2.3.

Total RNA (1 μg) was reverse transcribed in 40 μL final reaction volume using TaqMan Universal PCR Master Mix kit (Applied Biosystem) according to the manufacturer's instructions. The cDNA was synthesized at 48°C for 60 min and then stored at −20°C. Real-time PCR primer sequences were designed using Primer Express 3.0 software. Quantitative real-time polymerase chain reaction (qRT-PCR) was performed using SYBR green PCR Master Mix and Step ONE Real Time PCR System (Applied Biosystems) according to the manufacturer's instructions to measure *nanA* gene expression. *rpoA* amplifications were used as normalization controls. Each sample was analyzed three times to obtain average data. Relative transcript levels were calculated using the 2^−ΔΔCt^ formula (Ct = threshold cycle; Livak and Schmittgen, 2001).

### Production of recombinant NanA protein and polyclonal anti NanA antibodies

*E. coli* MV1161 was amplified from host DNA by polymerase chain reaction (PCR) using the following primers: FW AGCGGATCCATGGCAACGAATTT; RW AATAAGCTTTCACCCGCGCTCTT, where the recognition sites for BamHI and HindIII are underlined. To obtain the *nanA* gene, the PCR product was digested with the appropriate restriction enzyme and cloned into the BamHI and HindIII sites of the pET28a vector. The resulting plasmid contained the coding sequence for the recombinant NanA protein fused to a 6-histidine tag to facilitate protein purification.

The recombinant gene was expressed into the *E. coli* strain BL21. Cells were grown in LB medium at 37°C with 50 μg/mL kanamicin to an optical density of ~0.5 at 600 nm, and 0.1 mM isopropyl-beta-D-thiogalactopyranoside (IPTG) was added. The culture was grown for 3 h at 37°C for NanA production. Cells were harvested by centrifugation at 5000 rpm for 15 min at 4°C. The cellular pellet was resuspended in equilibration buffer (20 mM Na_2_HPO_4_, pH 7.4, 500 mM NaCl, 20 mM imidazole) containing 1 mM PMSF and disrupted by passage through a French Press.

The cell extract was centrifuged at 13000 rpm for 20 min at 4°C, to remove cell debris and the supernatant was filtered with a syringe-driven filter (0.22 μm) before protein purification. Soluble cell extract was loaded onto a His-Select Nickel Affinity Gel equilibrated with equilibration buffer and the bound protein was eluted with 500 mM imidazole in 50 mM phosphate buffer pH 7.4, 500 mM NaCl.

Protein concentration was estimated with Bradford reagent (Bio-Rad protein assay), protein purity was assessed by SDS-polyacrylamide gel electrophoresis (SDS-PAGE) and its primary structure verified by MALDI mapping strategy. Purified NanA protein was used to produce antiNanA antibodies by rabbit immunization (PRIMM, Milano, Italy).

### Western blot

*E. coli* cultures were grown at 37°C in the presence and in the absence of 0.04% MMS according to proteomics experiments. The cell pellets were lysed by French Press, resolved on a 12.5% SDS-PAGE gel and transferred onto a PVDF membrane. The membrane was blocked with 3% v/v milk in PBS buffer at room temperature for 1 h. The immunodetection was conducted by incubating the membrane with the anti-NanA antibodies (1:300, Primm, Milano Italy) for 18 h at 4° C under slow stirring. The membrane was then incubated with the secondary antibody conjugated to horseradish peroxidase (1:50000) for 1 h. Target proteins were visualized by ECL detection (Pierce).

### Static biofilm assays

A quantity of 2 mL of overnight bacterial cultures grown in LB + 1% glucose was added into 6-well flat bottomed polystyrene plate (Falcon). The plates were incubated at 37°C in aerobic conditions in the presence of 0.04% MMS. Growth was monitored by measuring the OD600, and after 24 h incubation the ability of the *E. coli* strain to adhere to the polystyrene plates was tested. The liquid medium was removed and the wells washed with sterile distilled water. The plates were then stained with 0.1% crystal violet for 20 min (O'Toole, 2011). Excess stain was rinsed off by placing the plate under running tap water. After the plates were air dried, the dye bound to the adherent cells was solubilized with 20% (v/v) glacial acetic acid and 80% (v/v) ethanol per well. The OD of each well was measured at 590 nm.

Biofilm assays on the complemented null NanA mutant strain were performed in the same conditions described above in the presence of 0.1 mM IPTG. Biofilm formation in the presence of a synthetic inhibitor was carried out on *E. coli* MV1161 strain grown in sterile 6-well flat-bottomed polystyrene plates treated with different concentrations of N-Acetyl-2,3-dehydro-2-deoxyneuraminic acid (DANA) ranging from 1 to 100 μg/mL. The plates were incubated aerobically at 37°C for 24 h and biofilm production was quantified as previously described.

### Invasion and adhesion assays on HeLa and Caco-2 eukaryotic cell lines

Bacteria from 18 h cultures in BHI broth, grown in the absence of MMS were further subcultured up to OD600 of 0.5 at 37°C with or without MMS 0.04%. HeLa cells, cultured in 24-well plates to obtain semi-confluent monolayers (1 × 10^5^ cells/well), were infected with 0.05 mL of logarithmically grown bacteria for 1 h at 37°C.

For the invasion assay, the monolayers were washed with PBS and treated with 0.5 mL of fresh medium containing 200 mg/mL of gentamicin for 1 h at 37°C to kill extracellular bacteria. Cells were then lysed by the addition of 0.025% Triton X-100 and plated on LB to count viable intracellular bacteria (Artini et al., 2012).

For the adhesion assay, loosely bound bacteria were removed from the cell monolayers by two washes with PBS. Cells were then lysed with 0.025% Triton X-100 and plated on LB agar to determine viable adherent bacteria. Adhesion efficiency was expressed as the percentage of the inoculated bacteria that adhered to HeLa cells.

Caco-2 intestinal cells were used for invasion and adhesion assays with AIEC LF82 strain (Darfeuille-Michaud et al., 2004). Caco-2 cells were seeded in a 12-well plate at a density of 4 × 10^5^ cells/well, let stand for 1 day, and medium (DMEM with low glucose supplemented with 10% FBS) replaced the day of infection. One mL of a 6 h-old LF82 culture (logarithmic phase) was harvested, and MMS at 0.04% was added for 20 min in one tube, while another was left non-treated as a control. After the induction step, both tubes were centrifuged at 3000 rpm for 5 min, and bacterial pellets resuspended in PBS. 10 μL of resuspended pellets were added to each well to obtain a MOI of bacteria/cells 10:1, and 12-well plates were incubated for 4 h at 37°C, 5% of CO_2_.

For the invasion assay, cells were washed three times with PBS and 2 mL of 22°C pre-warmed culture medium supplemented with 400 μg/mL of gentamicin were added to each well and the plate was incubated for 2 h at 37°C, 5% of CO_2_. Gentamicin was then removed washing three times with PBS and 1 mL of 1% Triton X-100 was added for 5 min during which the plates were gently swirled three times. Serial dilutions in PBS were performed till 10^−6^ and 100 μL of each dilution were plated onto TSA petri dishes (repeated three times). Colony-forming units (CFU) per mL were enumerated and the invasion index was expressed as the number of invasive bacteria per single Caco-2 cell.

For the adhesion assay, cells were washed three times with PBS and 1 mL of 1% Triton X-100 was added for 5 min, during which the plates were gently swirled three times. This concentration of Triton X-100 had no effect on bacterial viability for at least 30 min. Serial dilutions in PBS were performed till 10^−6^ and 100 μL of each dilution were plated onto TSA petri dishes (repeated three times). Colony-forming units (CFU) per mL were enumerated and the adhesion index was expressed as the number of adhesive bacteria per single Caco-2 cell.

Three biological replicates of both the invasion and adhesion assays were performed and the results expressed as mean ± standard error of the mean.

## Results

### DIGE experiments

**Figure 1:**
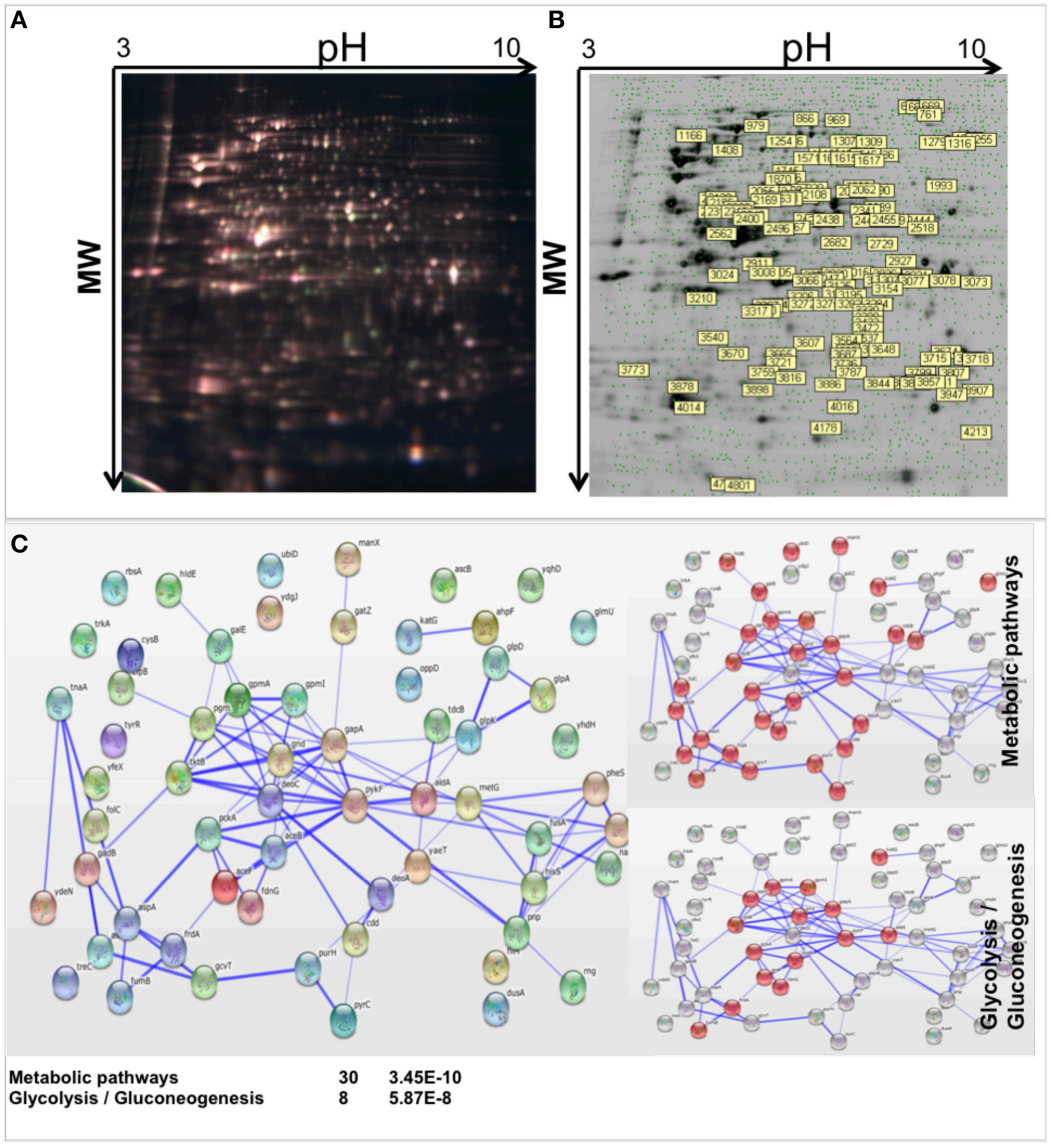
**Differential proteomic analysis of MMS treated MV1161 *E. coli*. (A)** Differential in Gel Electrophoresis (DIGE) of protein extracts from MV1161 *E. coli* in the absence and in the presence of MMS. Superimposed images of the individual fluorescent scans of analytical gels for the four analyzed biological replicates. **(B)** Semi-Preparative 2D-Gel Electrophoresis of protein extracts from MV1161 *E. coli*. The differentially expressed proteins in the presence of MMS submitted to mass spectrometric identification are labeled. **(C)** The network distribution of the 61 differentially expressed proteins according to STRING software. The top-ranked networks were in Metabolic Pathways, Glycolysis/Gluconeogenesis. Stronger associations are represented by thicker lines.

Differential in Gel Electrophoresis (DIGE) was used to examine the global effect of methylation stress on *E. coli*. Bacterial cells were grown in four replicates and each culture was divided into two aliquots, one of them was treated with 0.04% MMS and the other one was kept untreated and used as control. Protein lysates from MMS treated and untreated cells were extracted and fractionated by bidimensional electrophoresis using a 3–10 pH gradient. The relative quantitative analysis was performed so that only proteins up or down expressed in all four treated cell samples versus control were considered (Figure 1A). The corresponding preparative gel is reported in Figure 1B, where differently expressed proteins in the presence of MMS are labeled and were submitted to the identification procedure.

The spots of interest were picked, *in situ* hydrolyzed with trypsin and the resulting peptide mixtures analyzed by LC-MS/MS. Proteins were identified using the Mascot software. A total of 69 differentially expressed proteins were identified in MMS treated *E. coli* cells compared to controls, 61 down and 8 up expressed, and are reported in Table 1. Details of mass spectrometry analysis were reported in Table 1S of Supplemental Materials.

**Table 1:** **Differentially expressed proteins**.

**2^x^**	**n SPOT**	***p*-value**	**Protein description**	**Gene symbol**	**Swiss prot**
**METABOLIC PATHWAYS**					
−2.76	1545	0.0018	Transketolase 2	tktB	P33570
−1.82	1530	0.0049	Fumarate reductase flavoprotein subunit	frdA	P00363
−1.73	2441	0.0042	Bifunctional protein GlmU	glmU	P0ACC7
−1.67	3025	0.0023	Dihydroorotase	pyrC	P05020
−1.62	3317	0.0068	Cytidine deaminase	cdd	P0ABF6
−1.62	2006	0.0032	Fumarate hydratase class I, anaerobic	fumB	P14407
−1.56	2314	0.0039	aspartate ammonia-lyase	aspA	P0AC38
−1.53	3125	0.0028	PTS system mannose-specific EIIAB component	manX	P69797
−1.53	2041	0.0014	Bifunctional purine biosynthesis protein PurH	purH	P15639
−1.53	3125	0.0028	L-threonine dehydratase catabolic TdcB	tdcB	P0AGF6
−1.51	2562	0.0061	Thymidine phosphorylase	deoA	P07650
−1.39	3005	0.0041	Aspartate–ammonia ligase	asnA	P00963
−1.30	1408	0.0029	Catalase-peroxidase	katG	P13029
−1.30	2055	0.0057	3-octaprenyl-4-hydroxybenzoate carboxy-lyase	ubiD	P0AAB4
−1.30	2055	0.0057	Malate synthase A	aceB	P08997
−1.26	3070	0.0094	UDP-glucose 4-epimerase	galE	P09147
−1.25	2348	0.00189	Glycerol kinase	glpK	P0A6F3
−1.21	2467	0.0089	Bifunctional protein FolC	folC	P08192
−1.15	3787	0.0027	2,3-bisphosphoglycerate-dependent phosphoglycerate mutase	gpmA	P62707
+1.09	2400	0.0063	Glutamate decarboxylase beta	gadB	P69910
+1.09	2400	0.0063	Bifunctional protein HldE	hldE	P76658
−1.52	2050	0.00081	Phosphoenolpyruvate carboxykinase	pck	P22259
−1.40	1870	0.0015	Trehalose-6-phosphate hydrolase	treC	P28904
−1.79	3026	0.0012	Galactitol−1-phosphate dehydrogenase	gatD	P0A9S4
−1.65	2131	0.0032	2,3-bisphosphoglycerate-independent phosphoglycerate mutase	gpml	P37689
−1.30	2055	0.0057	Phosphoenolpyruvate carboxykinase [ATP]	pckA	P22259
+1.37	2105	0.0091	6-phospho-beta-glucosidase A	bglA	P24240
**GLYCOLYSIS/GLUCONEOGENESIS**					
−1.65	2131	0.0032	2,3-bisphosphoglycerate-independent phosphoglycerate mutase	gpml	P37689
−1.52	2050	0.00081	Phosphoenolpyruvate carboxykinase	pck	P22259
−1.51	2562	0.0061	6-phosphogluconate dehydrogenase	gnd	P00350
−1.48	3030	0.0068	Aldehyde reductase, NADPH-dependent	yqhD	Q46856
−1.41	3078	0.0024	Glyceraldehyde-3-phosphate dehydrogenase A	gapA	P0A9B2
−1.30	1408	0.0029	Dihydrolipoyllysine-residue acetyltransferase component of pyruvate dehydrogenase complex	aceF	P06959
−1.21	2467	0.0089	D-tagatose−1,6-bisphosphate aldolase subunit GatZ	gatZ	P0C8J8
−1.20	3721	0.0024	Deoxyribose-phosphate aldolase	deoC	P0A6L0
−1.15	3787	0.0027	2,3-bisphosphoglycerate-dependent phosphoglycerate mutase	gpmA	P62707
−1.12	2062	0.0069	Pyruvate kinase I	pykF	P0AD61
+1.24	2169	0.0053	Phosphoglucomutase	pgm	P36938
**AMINOACYL-tRNA BIOSYNTHESIS**					
−1.69	3047	0.0044	Phenylalanine tRNA synthetase, alpha subunit	pheS	P08312
−1.27	1307	0.0004	Threonine–tRNA ligase	thrS	P0A8M3
−1.19	3031	0.0071	tRNA-dihydrouridine synthase A	dusA	P32695
−1.17	2425	0.005	Histidine–tRNA ligase	hisS	P60906
+1.20	1571	0.0017	Methionine–tRNA ligase	metG	P00959
**MICROBIAL METABOLISM IN DIVERSE ENVIRONMENTS**					
−2.76	1545	0.0018	Transketolase 2	tktB	P33570
−1.85	761	0.0062	Formate dehydrogenase, nitrate-inducible, major subunit	fdnG	P24183
−1.56	2314	0.0039	Aldehyde dehydrogenase A, NAD-linked	aldA	P25553
**BIOSYNTHESIS OF SECONDARY METABOLITES**					
−5.78	3272	0.00024	N-acetylneuraminate lyase	nanA	P0A6L4
−1.68	3674	0.000046	Glucosamine-6-phosphate deaminase	nagB	P0A760
−1.72	2441	0.0042	Tryptophanase	tnaA	P0A853
−1.30	1408	0.0029	Polyribonucleotide nucleotidyltransferase	pnp	P05055
+1.09	2400	0.0063	Glutamate decarboxylase beta	gadB	P69910
**GLYCEROPHOSPHOLIPID METABOLISM**					
−1.65	2131	0.0032	Aerobic glycerol-3-phosphate dehydrogenase	glpD	P13035
−1.49	1963	0.0055	Anaerobic glycerol-3-phosphate dehydrogenase subunit A	glpA	P0A9C0
**TRANSPORTERS**					
−1.67	3025	0.0023	Oligopeptide transport ATP-binding protein OppD	oppD	P76027
−1.12	2062	0.0069	Ribose import ATP-binding protein RbsA	rbsA	P04983
+1.37	2105	0.0091	Potassium transporter peripheral membrane protein	trkA	P0AGI8
**BACTERIAL CHEMOTAXIS AND MOTILITY?**					
−1.32	3008	0.0066	Flagellar motor switch protein FliM	fliM	P06974
**OTHER**					
−1.62	3317	0.0068	Probable deferrochelatase/peroxidase YfeX	yfeX	P76536
−1.53	2041	0.0014	Ribonuclease G	rng	P0A9J0
−1.53	2041	0.0014	Transcriptional regulatory protein TyrR	tyrR	P07604
−1.38	3005	0.0041	Uncharacterized oxidoreductase YdgJ	ydgJ	P77376
−1.32	3008	0.0066	Aminomethyltransferase	gcvT	P27248
−1.32	3206	0.0033	Probable acrylyl-CoA reductase AcuI	acuI	P26646
−1.30	2055	0.0057	Uncharacterized sulfatase YdeN	ydeN	P77318
−1.26	3073	0.0072	HTH-type transcriptional regulator CysB	cysB	P0A9F3
−1.26	979	0.0049	Elongation factor G	fusA	P0A6M8
−1.26	979	0.0049	Chaperone protein ClpB	clpB	P63284
+1.51	2148	0.0031	Alkyl hydroperoxide reductase	ahpF	Q8XBT4
+1.45	2163	0.0077	Alkyl hydroperoxide reductase subunit F	ahpF	P35340
+1.11	1166	0.0056	Outer membrane protein assembly factor BamA	bamA	P0A940

### Biological network and functional annotation analysis

The network distributions of the 69 differently expressed proteins were explored using STRING software. The top-ranked networks were in Metabolic Pathways with a *p*-value of 3.45E-10, Glycolysis / Gluconeogenesis (*p* = 5.87E-8) and Microbial metabolism in diverse environments (*p* = 2.11E-7). Stronger associations are represented by thicker lines (Figure 1C).

Most of the identified proteins were under expressed with only a few found over expressed. The majority of the down expressed proteins belong to metabolic processes, suggesting a decrease in energy production. However, the most intriguing result in the presence of MMS was the sharp decrease in the expression of the N-acetylneuraminate lyase (NanA) an enzyme involved in sialic acid metabolism. Under methylating stress conditions, NanA expression was reduced by a factor of 2^6^, which corresponds to about 60 times decrease in protein concentration.

### Western blot analysis upon methylation stress

Proteomic data were independently confirmed by western blot analysis. Recombinant NanA protein bearing a N-terminal His tag was homologously produced in *E. coli* and purified by metal affinity chromatography. The recombinant protein was characterized by SDS-PAGE and its primary structure verified by MALDI mapping (Figure 1S of Supplemental Material). Specific anti NanA antibodies were then produced in rabbit, tested using different amounts of *E. coli* extracts and used to assess the expression of NanA under methylation stress conditions.

**Figure 2:**
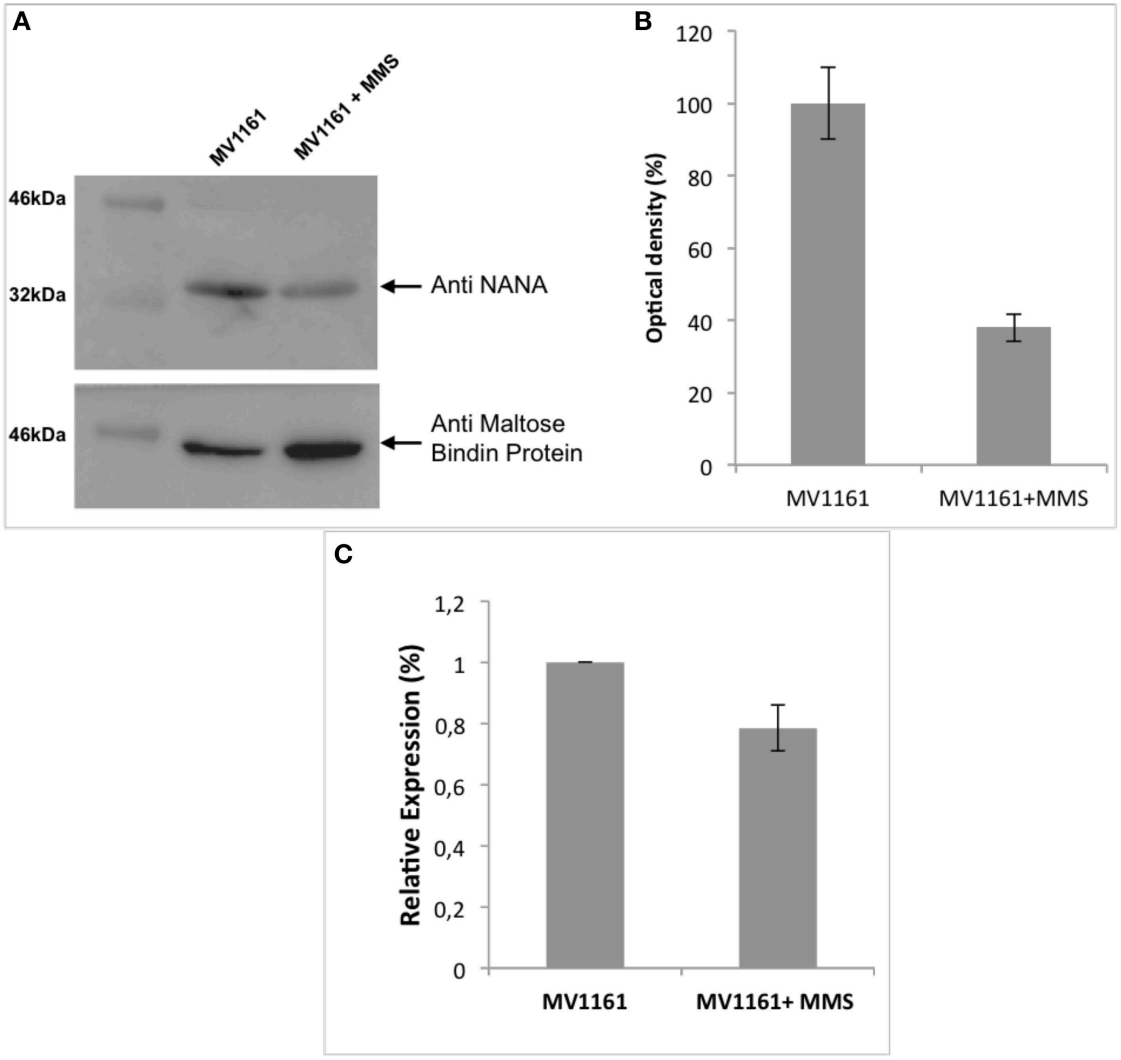
**Effect of methylation stress on expression levels of NanA. (A)** Western blot analysis of protein extracts from *E. coli* in the absence and in the presence of 0.04% MMS. **(B)** Densitometric analysis of the immunoblot in **(A)**. Protein content was normalized using the Maltose Binding Protein. Data are expressed as the percentage of relative expression and represent the mean ± SD of three replicates. **(C)** *nanA* gene expression levels measured by real time PCR assays in both MMS treated and untreated *E. coli* cells. Data are expressed as the percentage of relative expression and represent the mean ± SD of three replicates.

Figure 2A shows the western blot analysis of protein extracts from *E. coli* grown in the absence and in the presence of 0.04% MMS; the corresponding densitometric analysis is shown in Figure 2B. Protein content was normalized using the Maltose Binding Protein that was demonstrated to be unaffected by methylation stress by DIGE analysis. A strong decrease in the amount of the lyase was clearly detected upon methylation stress, thus confirming proteomic results.

### Effect of methylation stress on expression levels of nanA gene, biofilm formation and adhesion capability of non pathogenic *E. coli*

The significant down regulation of NanA was further investigated at mRNA level. Total RNA was extracted from *E. coli* cells either treated or untreated with 0.04% MMS demonstrating a high quality product.

Total RNA was reverse transcribed to obtain cDNA. Real time PCR assays were carried out to detect *nanA* gene expression levels in both MMS treated and untreated *E. coli* cells. Melting point curves displayed a single peak and any amplification of non-specific targets or primer dimers was not observed demonstrating the specificity of amplification. *rpoA* was used as housekeeping gene to normalize the threshold cycle values (Ct). Analyses were performed in triplicate.

The results of PCR amplification are shown in Figure 2C. Transcription of *nanA* gene was unaffected by MMS treatment showing a RNA amount almost similar to the untreated cells being the difference shown in Figure 2C not significant. Altogether these data suggested the occurrence of a negative translational control of *nanA* exerted by an unknown cellular mechanism in the presence of methylation stress.

Next, we explored the possibility that decreasing NanA levels upon exposure to methylating agents might also affect biofilm formation and adhesion abilities in the non-pathogenic MV1161 *E. coli* strain.

**Figure 3:**
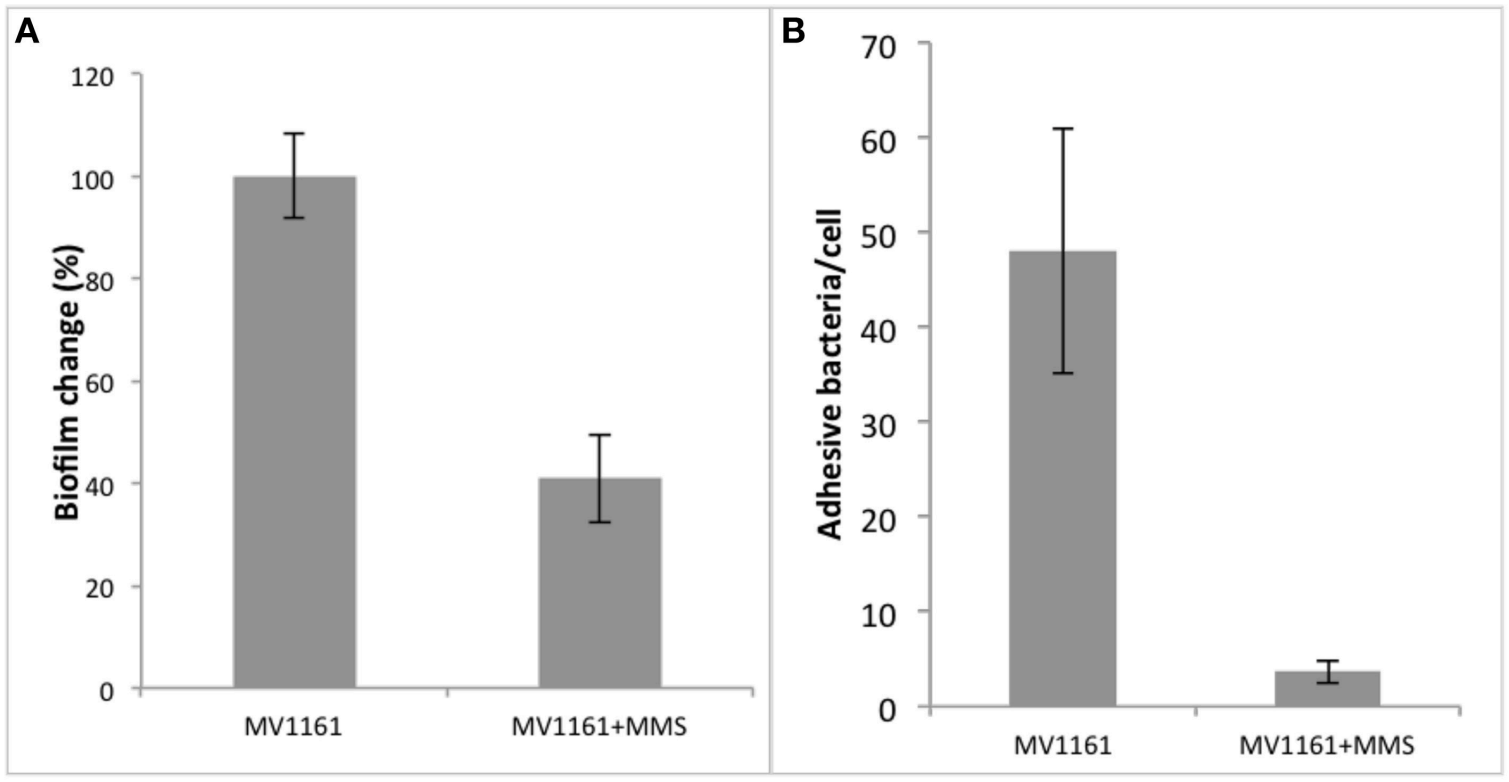
**Biofilm formation and adhesion capability of MMS treated non pathogenic *E. coli*. (A)** Biofilm formation of non pathogenic *E. coli* in the presence of MMS. Data are expressed as the percentage of crystal violet absorbance and represent the average of three independent experiments. **(B)** Adhesion capability of non-pathogenic *E. coli* to HeLa cells in the presence and in the absence of MMS. Data represent the average of three independent experiments.

*E. coli* cells were then grown in LB medium supplemented with 1% glucose either in the presence or in the absence of 0.04% MMS. As shown in Figure 3A, methylation stress decreased biofilm formation by about 60% compared to non-treated cells.

Since biofilm mechanisms are usually associated to cellular adhesion/invasion processes, we tested the effect of methylation stress on the capabilities of MV1161 to adhere to human HeLa cells. Following 0.04% MMS treatment, *E. coli* cells were subcultured and several different dilutions added to HeLa cells in order to establish the best bacterial/eukaryotic cells ratio. Analyses were performed in triplicate. Data obtained for one of the three cellular dilutions are reported in Figure 3B showing that *E. coli* adhesion on HeLa cells was drastically decreased in the presence of 0.04% MMS.

Since NanA expression was heavily decreased following MMS treatment, these data might suggest a possible involvement of this enzyme in the bacterial cohesive properties affecting the biological processes leading to biofilm formation.

### Investigation of NanA role in *E. coli* biofilm formation

**Figure 4:**
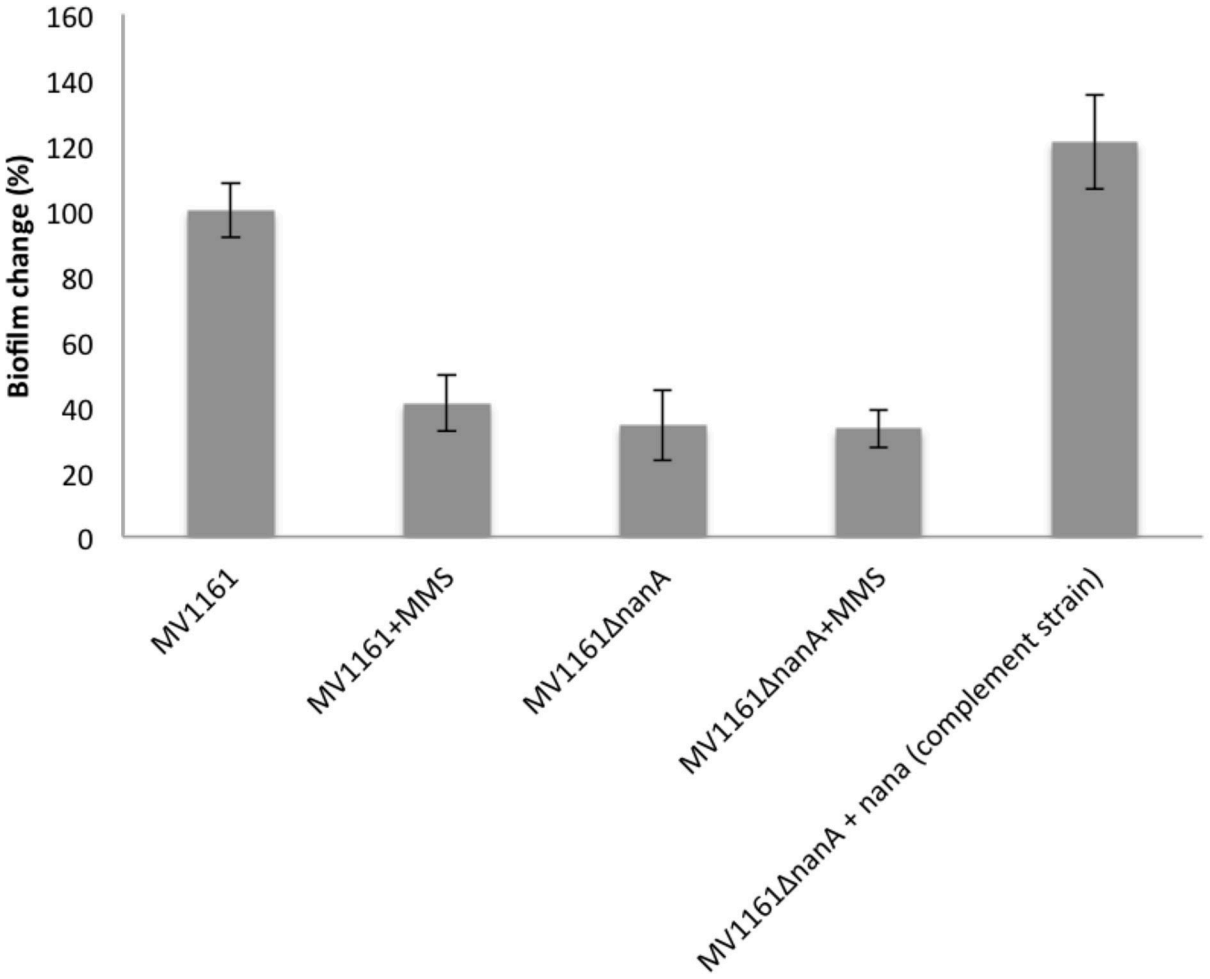
**Biofilm formation capability of the Δ*nanA* mutant *E. coli* strain**. Biofilm formation of both wild type and Δ*nanA* mutant *E. coli* strains in the absence and in the presence of 0.04% MMS, and of Δ*nanA* mutant complemented with the *nanA* gene. All data are expressed as percentage of crystal violet absorbance.

These observations prompted us to further investigate the putative role of NanA in the adhesion properties of *E. coli*. A null NanA mutant was constructed transforming electrocompetent *E. coli* MV1161 strain (Datsenko and Wanner, 2000). Biofilm formation experiments were carried out on Δ*nanA* mutant in comparison with MMS treated and untreated MV1161 *E. coli* cells. As reported in Figure 4, biofilm formation was drastically reduced in the Δ*nanA* mutant with the effect being very similar to that observed for the MMS treated MV1161 strain.

Next we checked the effect of MMS on the residual biofilm formation in the *nanA* mutant strain to investigate whether the observed decrease in the MMS treated cells and in the *nanA* mutant strain are both due to loss of NanA. Biofilm formation experiments were performed on Δ*nanA* mutant under methylation stress conditions in comparison with untreated MV1161 strain as control. Treatment with MMS did not reduce further the biofilm levels in the nanA mutant strain as shown in Figure 4, thus indicating that the two effects were not additive.

The biological implication of the lyase in biofilm formation was further supported by complementation assays. The Δ*nanA* mutant was transformed with a plasmid vector harboring a wild type copy of the gene, and the resulting complemented strain was used in biofilm assays as previously described. Figure 4 shows that biofilm production was greatly increased in the complement strain in comparison to MV1161 wild type strain, demonstrating that re-integration of the lyase NanA fully restored the ability of the mutant strain to adhere to abiotic surface.

**Figure 5:**
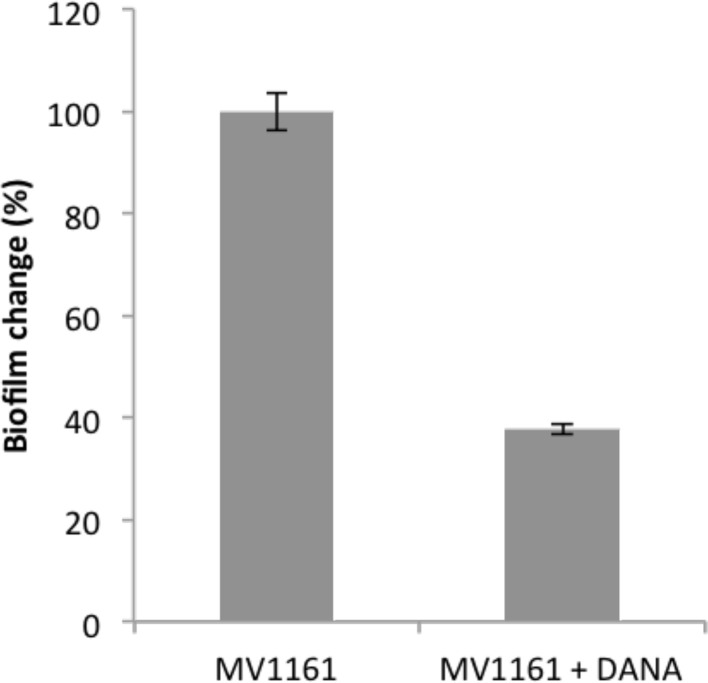
**Effect of DANA inhibitor on biofilm formation of MV1161 *E. coli* strain**. Biofilm formation of MV1161 *E. coli* strain in the absence and in the presence of DANA inibitor. Data are expressed as percentage of crystal violet absorbance.

The fundamental role of NanA in biofilm formation was finally assessed by performing biofilm experiments in the presence of N-Acetyl-2,3-dehydro-2-deoxyneuraminic acid (DANA). Although DANA is known as a sialidase inhibitor, we hypothesized that, being structurally related to sialic acid, it might also act as a competitive inhibitor of the lyase activity. Biofilm formation was performed in the presence of increasing DANA concentrations ranging from 1 to 100 μg/mL. Figure 5 shows biofilm production of *E. coli* MV1161 strain in the absence and in the presence of 50 μg/mL DANA inhibitor. From these experiments, we can infer that inhibition of lyase activity greatly affected the biofilm production properties of *E. coli* as demonstrated by the clear decrease in biofilm formation to a level very similar to that observed following MMS incubation.

### Effect of methylating agent on adhesion/invasion capabilities of pathogenic *E. coli* LF82 strain

In order to verify if MMS could affect adhesive and invasive properties of a pathogenic *E. coli*, we used the LF82 strain, an adherent-invasive *E. coli* (AIEC) pathogen associated with Crohn's disease development (Boudeau et al., 1999). This strain showed strong adhesive and invasive capabilities in intestinal cells and prolonged intracellular survival in macrophages, leading to enhanced pro-inflammatory cytokines release (Bringer et al., 2012).

**Figure 6:**
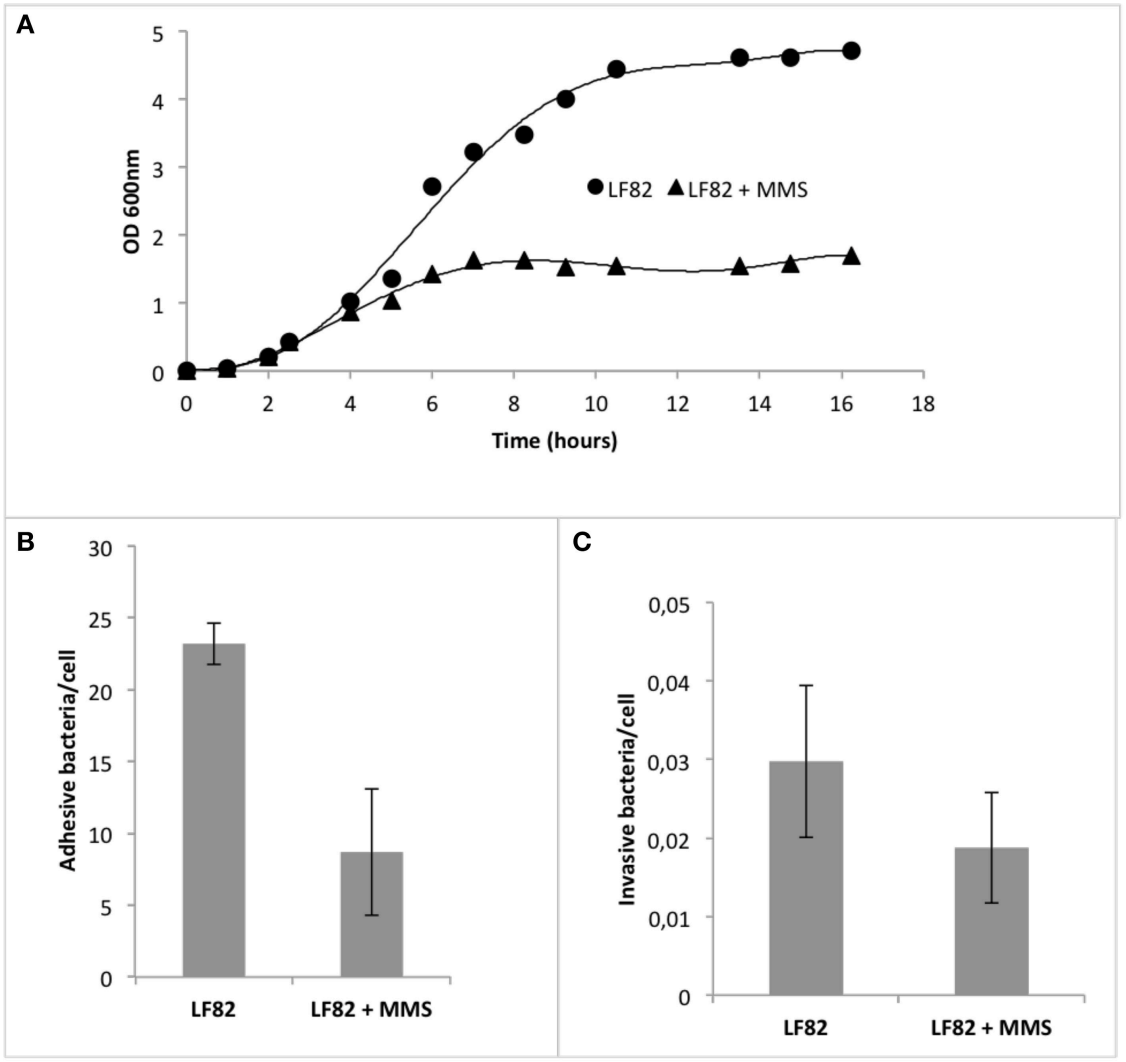
**Effect of methylation stress on pathogenic *E. coli* strain LF82 (AIEC)**. **(A)** Growth curves of AIEC strain LF82 in the presence (triangle) and in the absence (circles) of MMS. Superimposed to data points, for each growth curve, is the 4-parameter logistic regression following the formula *y* = *y*_0_ + *a*/[1+(*x*/*x*_0_)^*b*^], where *y*_0_ is the intercept with *y*-axis, *a* is the maximum value of *y*, *x*_0_ is the mid-point, and *b* is the curvature coefficient. **(B)** Adhesion indexes of LF82 strain on Caco-2 cells after 4 h infection in the presence and in the absence of MMS. Values are reported as the mean adhesive bacteria (± standard error of the mean) per single Caco-2 cell. **(C)** Invasion indexes of LF82 strain on Caco-2 cells after 4 h infection in the presence and in the absence of MMS. Values are reported as the mean adhesive bacteria (± standard error of the mean) per single Caco-2 cell.

As shown in Figure 6A, upon addition of 0.04% MMS, LF82 strain showed a decreased steady state level in the growth curve (OD_600LF82_ = 4.7 vs. OD_600LF82+MMS_ = 1.6), a different mid-point (*t*_mid−pointLF82_ = 5.9 h vs. *t*_mid−pointLF82+MMS_ = 3.8 h), and a diminished intrinsic growth rate in the logarithmic phase (*r*_LF82_ = 0.76 vs. r_LF82+MMS_ = 0.37). This result revealed that MMS affected LF82 growth curve, leading to the hypothesis of a parallel alteration in its adhesive and invasive properties.

To asses this point, we performed adhesion and invasion assays upon 4 h of LF82 infection on intestinal Caco-2 cells, a cell line deriving from gut epithelium, the initial target of LF82 adhesion and invasion, in the presence of MMS at concentrations of 0.04%, which did not affect the viability of the pathogenic strain (Figure 6B). Results showed that adhesion was diminished by 60.2% (Mann-Whitney, *p* = 0.0238), while invasion by 53.5% (Mann-Whitney, *p* = 0.0353; Figure 6C). Interestingly, MMS treatment led also to a diminished aggregative behavior of LF82, which usually aggregates within cell-to-cell junctions on Caco-2 monolayers (data not shown).

## Discussion

We have pursued a comprehensive investigation at the molecular level on the effect of sub-lethal concentration of MMS in *E. coli*. Using a differential proteomics approach we could establish that short-term exposure to MMS results in a general down regulation of protein expression. However, this effect does not seem to be due to non-specific inhibition of protein synthesis, since not all proteins are affected in the same way. A vast majority of the under expressed proteins belong to glycolysis and fatty acid degradation pathways, strongly suggesting a decrease in energy production. Thus, it might be tempting to speculate that reduction in energy flow, rather than DNA damage, might be responsible for growth inhibition by MMS observed in *E. coli*. Blocking energy generation in response to exposure to MMS might be an adaptive strategy to prevent further rounds of DNA replication, thus limiting DNA damage.

One of the most intriguing results of the proteomic experiment was the large decrease in the expression of the N-acetylneuraminate lyase NanA. This finding was also independently confirmed by western blot analysis using antibodies raised against a recombinant version of the NanA protein. NanA is the first enzyme of the canonical pathway of sialic acid catabolism including *nanA, nanK, nanE, nagA*, and *nagB* and catalyzes the aldolic cleavage of N-acetylneuraminic acid (sialic acid) to form pyruvate and N-acetyl-D-mannosamine (Vimr, 2013).

Sialic acid was reported to play a pivotal role in molecular recognition and, particularly in sialidase negative bacteria like *E. coli*, this amino sugar and its metabolites could be a key signal in cell-cell interactions (Sohanpal et al., 2004). Indeed, a correlation between sialic acid availability, biofilm formation and invasion of host cells in pathogenic bacteria has already been reported (Trappetti et al., 2009).

These observations prompted us to further investigate whether the decrease in NanA expression might also affect cellular adhesive properties and possibly the infection mechanism in commensal and pathogenic strains of *E. coli*. First we examined the transcription of the *nanA* gene in the presence of MMS by RT-PCR experiments showing that methylation stress did not affect mRNA production, suggesting that other unknown mechanisms might be responsible for the low amount of the protein. Experiments on the elucidation of these molecular mechanisms are currently being carried out.

Next we demonstrated that exposure to MMS diminished biofilm formation of about 60% in the MV1161 laboratory strain. Analogously, *E. coli* adhesion to HeLa cells resulted drastically decreased in the presence of MMS, indicating that a correlation exists between methylation stress and cellular adhesive properties. Interestingly, MMS treatment decreased by almost 50% the adhesive properties of LF82, a pathogenic *E. coli* strain associated to Crohn disease, with strong adhesive and invasive capabilities in intestinal cells (Miquel et al., 2010).

These results led us to speculate that inhibition of cell adhesion might be related to downregulation of the NanA lyase, in line with the involvement of sialic acid in biofilm formation and adhesion properties. To investigate this point, we produced an *E. coli* mutant strain lacking the *nanA* gene. We only constructed the nanA mutant in the MV1161 background, as attempts to create mutants in the LF82 strain have been so far unsuccessful due to its lower amenability to genetic manipulation. The Δ*nanA* mutant showed a phenotype nearly superimposable to the control MV1161 *E. coli* under methylation stress conditions. Moreover, no further reduction in biofilm formation was detected when the Δ*nanA* mutant was treated with MMS, suggesting that impairment of cell-cell interactions by methylation stress is only related to the lyase biological activity.

This hypothesis was further supported by both complementation assays and using a competitive synthetic inhibitor. Re-integration of the *nanA* gene within the Δ*nanA* strain resulted in the rescue of bacterial phenotype completely restoring the ability of the mutant strain to produce biofilm. Adhesion/invasion assays on both the mutant and the complemented strain were not performed since both strains derived from the MV1161 non pathogenic *E. coli* species devoted of invasion abilities Finally, in the presence of the DANA inhibitor, biofilm formation was greatly diminished in MV1161 *E. coli* strain decreasing to a level very similar to that observed under methylation stress conditions.

Sialic acid is widely recognized as a signaling molecule playing a role in the mechanism of biofilm formation, colonization and host invasion at different extent (Sohanpal et al., 2004; Trappetti et al., 2009). Nontypeable *H. influenzae* (NTHI) produces a lipooligosaccharide decorated with sialic acid due to the activity of a number of genes responsible for carbohydrate biosynthesis and assembly (Greiner et al., 2004). A causal association between sialic acid and pneumococcal biofilm formation *in vitro* was also demonstrated supporting the hypothesis that free sialic acid is a trigger that converts a harmless colonizing pneumococcus into an invasive pathogen. Finally, sialic acid and its metabolites GlcNAc and GlcNAc-6-P were also found involved in regulatory mechanisms affecting *E. coli* type I fimbriae expression, structure required for bacterial adhesion to host cells (Sohanpal et al., 2004).

These results confirmed the hypothesis that impairment of biofilm formation under methylation stress involves downexpression of NanA and underlined the importance of sialic acid metabolism to promote/prevent cell-cell interactions.

## Author contributions

PD and MC: Substantial contributions to the conception of the work AND Drafting the work. ADS and MS: the acquisition of data for the work. VI, SS, RP, LS, and MA: analysis of data for the work. PL, ER, AP: interpretation of data for the work. AD: Drafting the work, revising it critically for important intellectual content AND Final approval of the version to be published.

### Conflict of interest statement

The authors declare that the research was conducted in the absence of any commercial or financial relationships that could be construed as a potential conflict of interest.

## Supplementary material

Figure 1S **Purification of His Tagged NanA protein. (A)** Nickel Affinity chromatography of the *E. coli* expressed His-tagged NanA protein. **(B)** SDS-PAGE analysis of the purified recombinant NanA protein. Lane 1: total *E. coli* protein extrcts; lane 3: unbound proteins: lane 9–14: Nickel Affinity chromatography fractions eluted with 500 mM imidazole.

Table 1S **Details of tandem mass identification**.
