# Erratum: Exposure of *E. coli* to DNA-Methylating Agents Impairs Biofilm Formation and Invasion of Eukaryotic Cells via Down Regulation of the N-Acetylneuraminate Lyase NanA

**DOI:** 10.3389/fmicb.2016.00383

**Published:** 2016-03-16

**Authors:** Frontiers Production Office

**Affiliations:** Frontiers Production OfficeLausanne, Switzerland

**Keywords:** DNA alkylation, adaptive response, NanA lyase, comparative proteomics, biofilm formation, virulence, adherent invasive *Escherichia coli* (AIEC)

Reason for Erratum:

Due to a typesetting error, the name of the author Anna Teresa Palamara was reported as Annateresa Palamara and the citation as Palamara A instead of Palamara AT.

The original article has been updated and the publisher apologizes for this mistake.

This error does not change the scientific conclusions of the article in any way.

