# Supplementary figures and images for: Exposure of E. coli to DNA-Methylating Agents Impairs Biofilm Formation and Invasion of Eukaryotic Cells via Down Regulation of the N-Acetylneuraminate Lyase NanA
Source: Front Microbiol. 2016 Feb 11;7:147. doi: 10.3389/fmicb.2016.00147 (PMC4749703; doi:10.3389/fmicb.2016.00147)

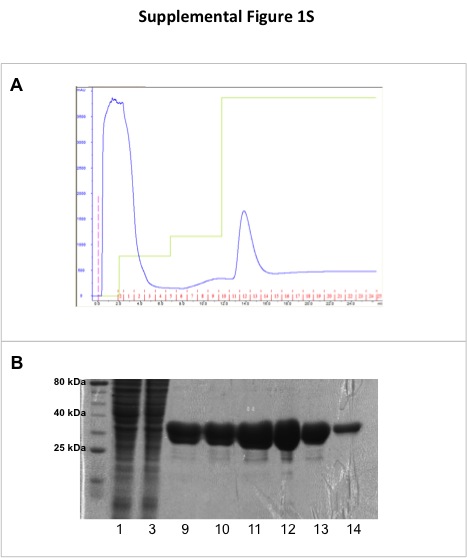

Supplement: Figure 1S — Purification of His Tagged NanA protein. (A) Nickel Affinity chromatography of the E. coli expressed His-tagged NanA protein. (B) SDS-PAGE analysis of the purified recombinant NanA protein. Lane 1: total E. coli protein extrcts; lane 3: unbound proteins: lane 9–14: Nickel Affinity chromatography fractions eluted with 500 mM imidazole. [file Image1.JPEG]
